# Global Prevalence of Zika and Chikungunya Coinfection: A Systematic Review and Meta-Analysis

**DOI:** 10.3390/diseases12020031

**Published:** 2024-01-31

**Authors:** Saleh Ahmed, Shabiha Sultana, Shoumik Kundu, Sayeda Sadia Alam, Tareq Hossan, Md Asiful Islam

**Affiliations:** 1 Center for Biotechnology and Genomic Medicine, Medical College of Georgia, Augusta University, Augusta, GA 30912, USA; 2 Department of Cellular Biology and Anatomy, Medical College of Georgia, Augusta University, Augusta, GA 30912, USA; 3 Department of Chemistry and Biochemistry, Texas Tech University, 2500 Broadway St., Lubbock, TX 79409, USA; 4 Department of Biochemistry and Molecular Biology, Jahangirnagar University, Savar, Dhaka 1342, Bangladesh; 5 Department of Internal Medicine, Division of Oncology, Washington University School of Medicine in St. Louis, St. Louis, MO 63110, USA; 6 WHO Collaborating Centre for Global Women’s Health, Institute of Metabolism and Systems Research, College of Medical and Dental Sciences, University of Birmingham, Birmingham B15 2TT, UK

**Keywords:** zika, chikungunya, coinfection, global prevalence, systematic review, meta-analysis

## Abstract

Zika virus (ZIKV) and chikungunya virus (CHIKV) are arthropod-borne viruses with significant pathogenicity, posing a substantial health and economic burden on a global scale. Moreover, ZIKV-CHIKV coinfection imposes additional therapeutic challenges as there is no specific treatment for ZIKV or CHIKV infection. While a growing number of studies have documented the ZIKV-CHIKV coinfection, there is currently a lack of conclusive reports on this coinfection. Therefore, we performed a systematic review and meta-analysis to determine the true statistics of ZIKV-CHIKV coinfection in the global human population. Relevant studies were searched for in PubMed, Scopus, and Google Scholar without limitation in terms of language or publication date. A total of 33 studies containing 41,460 participants were included in this meta-analysis. The study protocol was registered with PROSPERO under the registration number CRD42020176409. The pooled prevalence and confidence intervals of ZIKV-CHIKV coinfection were computed using a random-effects model. The study estimated a combined global prevalence rate of 1.0% [95% CI: 0.7–1.2] for the occurrence of ZIKV-CHIKV coinfection. The region of North America (Mexico, Haiti, and Nicaragua) and the country of Haiti demonstrated maximum prevalence rates of 2.8% [95% CI: 1.5–4.1] and 3.5% [95% CI: 0.2–6.8], respectively. Moreover, the prevalence of coinfection was found to be higher in the paediatric group (2.1% [95% CI: 0.0–4.2]) in comparison with the adult group (0.7% [95% CI: 0.2–1.1]). These findings suggest that the occurrence of ZIKV-CHIKV coinfection varies geographically and by age group. The results of this meta-analysis will guide future investigations seeking to understand the underlying reasons for these variations and the causes of coinfection and to develop targeted prevention and control strategies.

## 1. Introduction

Zika virus (ZIKV) and chikungunya virus (CHIKV) were first identified in Uganda and Tanzania in 1947 and 1952, respectively, and subsequently spread to other parts of the world [1,2,3,4]. In recent years, there has been a notable increase in the occurrence of epidemics caused by ZIKV and CHIKV on a global scale. These epidemics have resulted in substantial levels of mortality and morbidity [5,6]. According to the published data, it has been suggested that ZIKV and CHIKV were responsible for an annual average loss of more than 106,000 and 44,000 disability-adjusted life years (DALYs), respectively, during the period spanning from 2010 to 2019 [7]. Several factors, including climate change, urbanization, and increased travel activities, have caused the drastic amplification of these viruses, primarily in tropical and subtropical regions, but also in temperate zones [8,9,10,11].

ZIKV is classified as a tiny, enveloped, positive sense, single-stranded RNA virus which belongs to the Flavivirus genus [12,13,14,15]. As of July 2019, a total of 87 nations worldwide have documented indigenous transmission of ZIKV through mosquitoes. These cases are dispersed throughout four of the six regions recognised by the World Health Organization (WHO) [12]. Determining the accurate prevalence of ZIKV cases is challenging due to the presence of misclassification and underreporting, which introduces uncertainties regarding the reliability of the available data. The primary mode of transmission of ZIKV is the bite of infected *Aedes aegypti* mosquitoes [12,16]. Additionally, the virus can be transmitted through non-vector modes, including sexual intercourse, blood transfusion, saliva, from a mother to her foetus during pregnancy and childbirth, tissue, and organ transplantation as well as laboratory exposures [16,17,18,19,20]. The infection caused by ZIKV frequently manifests as asymptomatic, with approximately 75–80% of individuals not displaying any noticeable symptoms. However, those who do exhibit symptoms typically experience a mild sickness following an incubation period ranging from 3 to 12 days [19,20,21]. The primary manifestations of ZIKV infection include elevated body temperature, inflammation of the conjunctiva, rash, joint and muscle pain, headache, and in some instances, gastrointestinal disturbances. Moreover, ZIKV infection has been linked to neurological syndromes, including Guillain–Barré syndrome (GBS), transverse myelitis, and encephalitis [20,22,23].

CHIKV is categorised as an encapsulated, positive-sense, single-stranded RNA virus that belongs to the Alphavirus genus [24]. As in ZIKV cases, the issue of underreporting and inaccurate diagnosis has posed a significant challenge in establishing an accurate annual worldwide record of chikungunya infections. According to the European Centre for Disease Prevention and Control (ECDC), there have been an estimated 440,000 chikungunya virus disease (CHIKVD) cases and over 350 fatalities reported on a global scale until 30 September 2023 [25]. A total of 24 countries have reported cases of CHIKVD that derived from the continents of Asia (4), Africa (4), and the Americas (16). The majority of the countries that have reported a significant burden of CHIKVD are primarily situated in the Americas, specifically in South and Central America [25]. Like ZIKV, the primary mode of transmission of CHIKV to human beings is through the bites of infected mosquitoes, specifically the *Aedes aegypti* and *Aedes albopictus* species [26]. In addition, CHIKV can also be transmitted from mother to child [27]. CHIKV has the potential to induce clinical manifestations that are similar to other arbovirus-infection-related diseases. The disease is characterised by acute-phase manifestations such as rash, fever, and myalgia. However, the primary distinguishing feature of the condition is the presence of severe arthralgia, which has the potential to persist as a chronic condition [28].

The arboviruses are transmitted by the same vectors, and their simultaneous circulation is observed in numerous places worldwide [29,30,31,32,33,34,35]. Moreover, there have been earlier reports of concurrent infections, some of which have been linked to mortality [36,37]. It is important to enhance our understanding of the transmission dynamics of numerous arboviruses, as sequential infections and coinfections may contribute to the development of severe clinical symptoms [38]. Furthermore, the potential for misdiagnosis leading to severe health implications is heightened due to the occurrence of cross-reactivity and similarity in symptoms, such as fever, headache, rash, muscle pain, and arthralgia, with other arboviruses [38,39,40]. For example, misdiagnosis of ZIKV with CHIKV might potentially lead to significant consequences, as ZIKV infection has been linked to the development of Guillain–Barré syndrome (GBS), a serious and life-threatening condition of neuropathy. The possibility of such serious implications highlights the necessity for precise diagnosis methods. Given the challenges of identifying arboviral infections based solely on clinical observations, especially in regions where multiple viruses are circulating simultaneously, laboratory testing is necessary to ensure correct identification of these viral pathogens. In addition, only diagnostic techniques may identify simultaneous arboviral infections, which often happen during concurrent outbreaks and can have significant consequences for clinical results. Unfortunately, laboratory services that are efficient and reliable are not easily accessible in the majority of outpatient and urgent care units located in tropical and subtropical nations [41]. With no specific treatment and vaccine availability against ZIKV and CHIKV, current treatment focuses on the management of associated symptoms, like fever, pain, and headache, by taking pain relievers and taking sufficient rest, oxygen, and fluids. Moreover, prevention or control strategies, including installing window and door screens, employing air conditioning, utilizing insect repellent, minimizing mosquito bites throughout the day, and eliminating household debris and water containers, would help to reduce the spread of infection [42,43]. Thus, it is crucial to know the frequency of arboviruses such as ZIKV-CHIKV coinfection, so as to better manage their associated diseases. Although there are several sporadic reports on the co-occurrences of ZIKV and CHIKV, the pooled prevalence of such coinfection remains undetermined on a national, regional, and worldwide scale. Meta-analysis is one of the effective approaches by which to assess the frequency of coinfection in different populations and identify risk factors, including age, sex, socioeconomic status, and geographic location. The primary objective of this systematic review and meta-analysis is to establish an impartial foundation of data regarding the geographic prevalence of this coinfection among the human population. Therefore, this work presents a comprehensive analysis of the worldwide occurrence of ZIKV and CHIKV coinfection by the utilization of a meta-analytical approach.

## 2. Materials and Methods

### 2.1. Guideline and Protocol

Following Preferred Reporting Items for Systematic Reviews and Meta-analyses (PRISMA) standards [44], a systematic review and meta-analysis of published studies reporting instances of coinfection with the ZIKV and CHIKV worldwide was carried out. The research protocol was submitted to PROSPERO and given the registration number CRD42020176409.

### 2.2. Literature Search Strategy

Without placing any limitations on the time range or language, systematic searches were carried out across three electronic databases: PubMed, Scopus, and Google Scholar. The searches were last updated in June 2023. The search strategy employed a combination of relevant keywords to investigate the worldwide impact of zika and chikungunya coinfection. Because the aim of the study was to specifically investigate the simultaneous presence of both ZIKV and CHIKV in individuals, the search was conducted exclusively for coinfection of ZIKV-CHIKV. To find the highest possible number of articles, the following predefined search terms were used: “Zika”, “ZikV”, “Chikungunya”, “CHIK”, “CHIKV”, “co-infection” “co-infections”, “coinfection”, “coinfections”, “co-infected”, “coinfected”, “cocirculation” OR “co-circulation”, “concurrent”, “simultaneous”, “simultaneously”, “double-infected”, “dual”, “infection”, “infections”, “arbovirus”, “vector-borne”, “prevalence”, “seroprevalence”, “burden”, “epidemiology”, “epidemiological”, “epidemic”, “endemic”, “outbreak” and additional phrases coupled with Boolean operators AND and OR. Additionally, manual searches were conducted in the reference lists of the included studies. Detailed search strategies for the three different databases are listed in Table S1.

### 2.3. Eligibility Criteria

This study consisted of prospective and retrospective studies examining the prevalence of ZIKV-CHIKV coinfection within a specific population. The following types of literature were excluded from our study: (1) case reports, opinions, perspectives, book chapters, reviews, and editorials; (2) studies involving non-human subjects; (3) articles for which complete access to the full text was not available; (4) studies lacking clear or comprehensive data on ZIKV-CHIKV coinfection; and (5) studies relying on self-reported cases of infection rather than laboratory-based confirmation of the diseases.

To establish a comprehensive and reliable search methodology, the references of the study in question were thoroughly examined and evaluated. The EndNote X8 software was implemented to eliminate duplicate studies. In order to identify suitable studies, four authors (S.A., S.S., S.S.A. and S.K.) thoroughly evaluated the articles of interest. This evaluation involved an initial screening based on the title and abstract, followed by a comprehensive assessment of the full-text articles. Disputes regarding the concept of inclusion were effectively addressed through a process of discussion and adjudication, involving the participation of an additional two authors (M.A.I. and T.H.). The focus of this study was on the human population regardless of sex, age, or geographic location.

### 2.4. Data Extraction and Quality Assessment

An Excel file with pre-defined fields was used to extract data. Four authors (S.A., S.S., S.S.A. and S.K.) independently extracted the following information from the eligible studies: the first author’s last name, the year of publication, the time the samples were taken, the nation where the samples were taken, the study’s design, the total number of participants enrolled, the split between male and female participants, the participants’ ages, and the laboratory technique used to diagnose ZIKV-CHIKV coinfection. 

The quality of the included studies was assessed by two writers (S.A. and S.S.A.) independently using the Joanna Briggs Institute (JBI) critical evaluation technique [45], specifically designed for ZIKV-CHIKV coinfection prevalence studies (Table S2). The quality of the studies was classified based on their overall score as “poor quality” (<50%), “moderate quality” (50–70%), and “high quality” (>70%) [46].

A funnel plot was constructed to evaluate publication bias by plotting prevalence estimates against their corresponding standard errors. The Egger’s test was employed to validate the asymmetry of the funnel plot, where a *p*-value of <0.05 was deemed to be statistically significant.

### 2.5. Data Synthesis and Statistical Analysis

The random-effects model of restricted maximum likelihood (REML) was utilised to obtain the pooled prevalence and 95% confidence interval (CI) by compiling prevalence data and sample sizes from individual studies. The REML model assumes that there is a distribution of true prevalences across studies, considering both sampling error and significant variations between studies. The heterogeneity among studies was evaluated by employing the *I*^2^ statistic, where a value greater than 75% was considered indicative of significant heterogeneity. Furthermore, the Cochran’s Q test was employed to determine the statistical significance of heterogeneity [47,48]. In order to acquire prevalence estimates at national and regional levels and evaluate the factors contributing to variation, subgroup analysis was performed based on country, region, and age group (adult or, paediatric). Subgroup analysis was performed only on those groups which contain at least two studies. To determine the outlier studies, a Galbraith plot was also constructed to calculate the incidence of ZIKV–CHIKV coinfection.

The metaprop codes in the meta (version 6.1-0) and metafor (version 3.8-1) packages of R (version 4.2.2) and the RStudio environment (version 1.2.5033) were used to undertake the analyses and visualizations [49].

## 3. Results

### 3.1. Selection of the Relevant Studies

**Figure 1:**
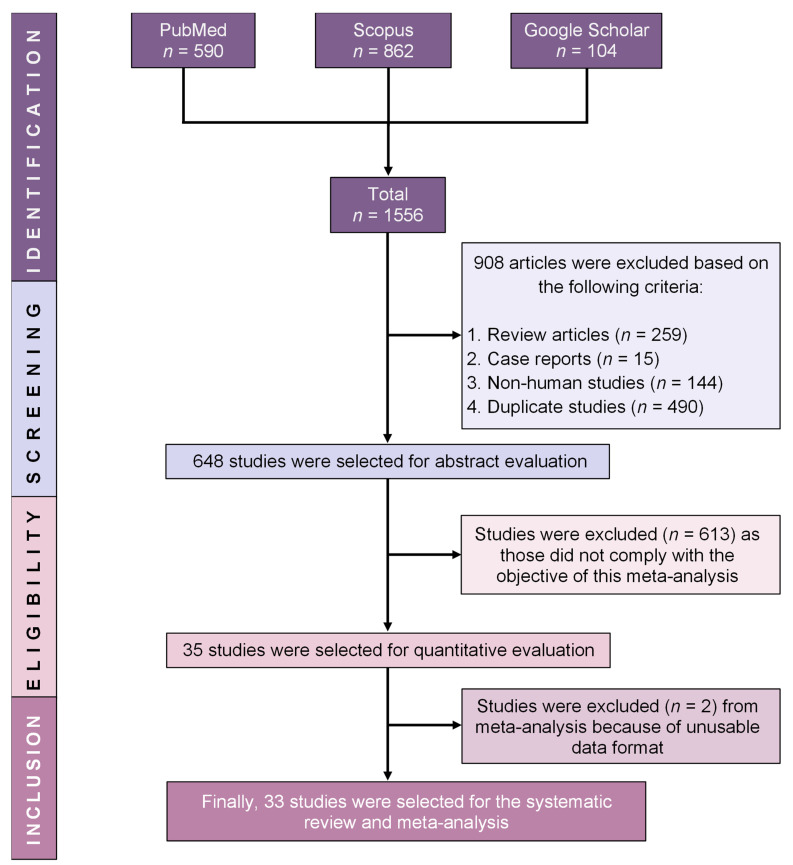
PRISMA diagram of study selection process.

Initially, the investigation of three electronic databases, including PubMed, Scopus, and Google Scholar, revealed an overall count of 1556 records. A total of 648 articles were subjected to screening based on their title and abstract after the elimination of duplicate articles, review articles, case reports, and non-human studies. The complete texts of the remaining articles were subsequently evaluated to determine their eligibility. After careful investigation of full texts, a further 615 research articles were removed from the study due to factors including unusable data formats and instances of non-compliance with the objective of this study. Finally, a total of 33 articles were identified as eligible and incorporated into the qualitative synthesis and meta-analysis. The comprehensive selection procedure is outlined in Figure 1.

### 3.2. Major Features of the Included Studies

Of the 33 cross-sectional studies with a sample size of 41,460, a total of 346 ZIKV–CHIKV coinfection cases were documented. Approximately 70% of the studies included in this meta-analysis originated from Brazil, while data were gathered from a total of 11 nations across the world. Participants of the included studies were both male and female as well as paediatrics and adults. Different techniques including RT-PCR, conventional PCR, ELISA, and immunoassay, and rapid test kit, were used to determine the coinfection of ZIKV and CHIKV. A comprehensive description of the key features of the included studies is shown in Table 1.

### 3.3. Major Outcomes

**Figure 2:**
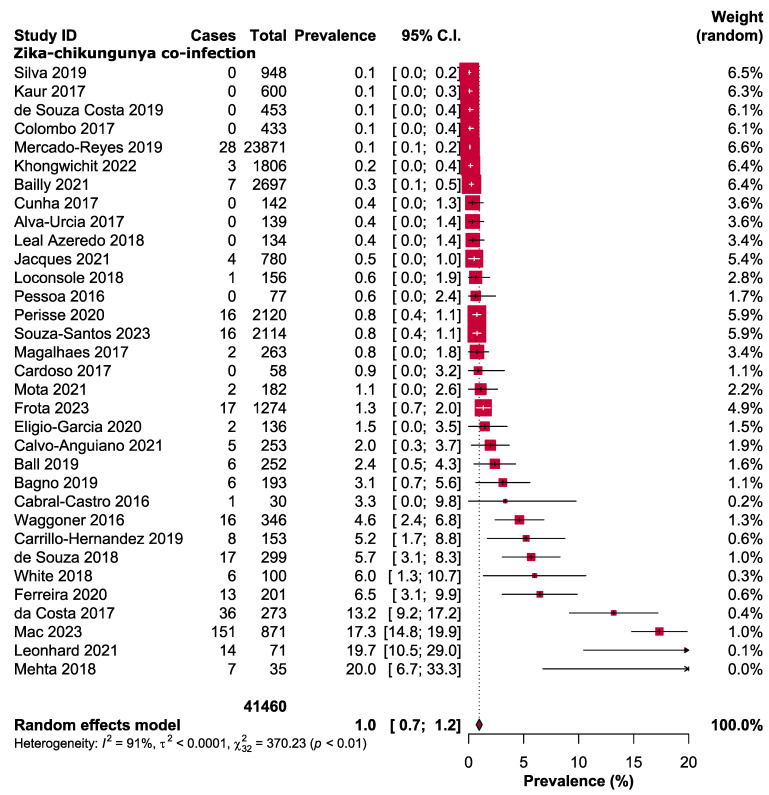
Pooled prevalence of ZIKV-CHIKV coinfection [11,31,32,33,34,35,36,50,51,52,53,54,55,56,57,58,59,60,61,62,63,64,65,66,67,68,69,70,71,72,73,74,75].

The overall prevalence of ZIKV-CHIKV coinfection was found to be 1.0% [95% CI: 0.7–1.2] (Figure 2). Moreover, subgroup analysis was performed to further investigate the studies based on the countries, subcontinents, and age groups of the participants. Considering the subcontinents of included studies, the coinfection frequency was highest in North America (Mexico, Haiti, and Nicaragua) with a rate of 2.8% [95% CI: 1.5–4.1], whereas the lowest prevalence was observed in Asia at 0.1% [95% CI: 0.0–0.3] (Table 2 and Figure S1). The prevalence estimates for coinfection, when sorted by countries, were as follows: Haiti had the highest prevalence at 3.5% [95% CI: 0.2–6.8], followed by Colombia at 2.4% [95% CI: 0.0–7.3], Mexico at 1.8% [95% CI: 0.5–3.1] and Brazil at 1.0% [95% CI: 0.6–1.4] (Table 2 and Figure S1). Based on the age groups, the coinfection rate was found to be higher in paediatrics (2.1% [95% CI: 0.0–4.2]) in comparison with adults (0.7% [95% CI: 0.2–1.1]) (Table 2 and Figure S1).

### 3.4. Publication Bias and Quality Assessment

**Figure 3:**
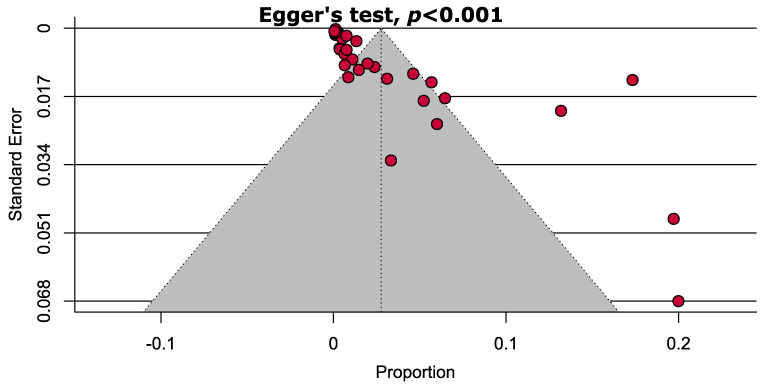
Funnel plot representing significant publication bias estimating the prevalence of ZIKV-CHIKV coinfection.

Quality assessment of the studies that were included in this systematic review and meta-analysis is outlined in Table S2. In summary, all of the cross-sectional studies included in this analysis were classified as high quality (67%) or moderate quality (33%). No low-quality studies were observed in this meta-analysis. There is a strong publication bias in the estimation of ZIKV-CHIKV coinfection according to the findings from the funnel plot and Egger’s test (*p* < 0.001) (Figure 3).

### 3.5. Outlier and Sensitivity Analysis

**Figure 4:**
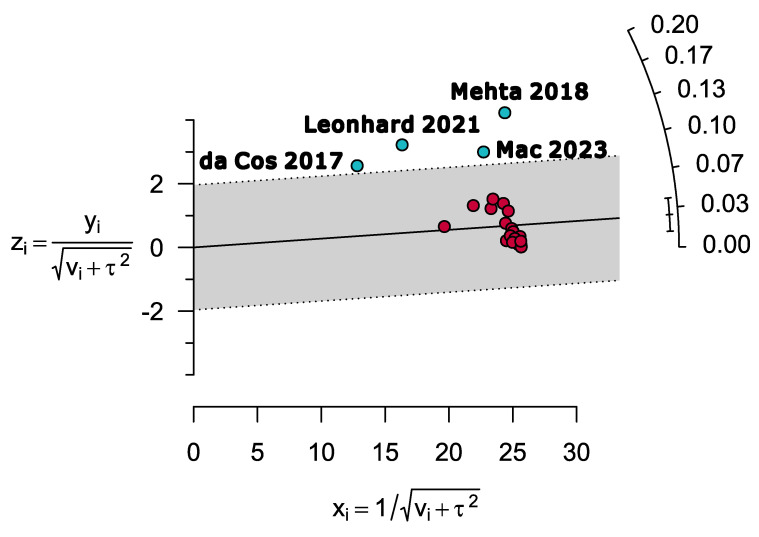
Galbraith plot representing four outlier studies estimating the prevalence of ZIKV-CHIKV coinfection [32,54,65,71].

Visual inspection of the Galbraith plot represented four outlier studies. The outlier studies were identified as Mac 2023 [32], Leonhard 2021 [54], Mehta 2018 [65], and da Costa 2017 [71] (Figure 4).

Sensitivity analyses were conducted to assess the incidence of ZIKV-CHIKV coinfection through the exclusion of studies with small sample sizes (n < 100) as well as low and moderate quality studies. Sensitivity analyses showed a prevalence of 0.9% [95% CI: 0.6–1.2] ZIKV-CHIKV coinfection upon exclusion of small studies, whereas only high quality studies revealed the prevalence of 0.6% [95% CI: 0.3–0.8] (Figure S2). Overall, the exclusion of small studies and of low and medium quality studies did not cause significant changes in the pooled prevalence of ZIKV-CHIKV coinfection.

## 4. Discussion

In recent decades, there have been multiple occurrences in which alphavirus, ZIKV, and flavivirus, CHIKV, outbreaks have been documented across various regions worldwide, specifically in urban settings with tropical/subtropical climates [70,72,76,77,78,79,80,81,82,83,84,85,86,87,88]. The global distribution and ongoing global expansion of the vectors, namely *Aedes aegypti* and *Aedes albopictus*, have a significant impact on the disease burden [89,90,91]. The expansion of the *Aedes aegypti* and *Aedes albopictus* vectors from their original geographical location to other regions has been facilitated by various contributing factors, including globalization, urbanization, and climate change [92,93]. Nevertheless, these disorders are not frequently examined as a component of standard laboratory diagnosis in countries with limited resources. Despite the significant disease burden associated with mono-infections of ZIKV and CHIKV, there exists a wide range of documented cases of concurrent infections [11,29,55,61,62,94]. It is crucial to consider the presence of coinfections, given the absence of targeted therapeutic interventions and preventative vaccines for these infectious diseases. Given these facts, we conducted a meta-analysis to examine the global prevalence and distribution of ZIKV-CHIKV coinfection. To the best of our understanding, this study represents the first comprehensive examination and synthesis of existing literature pertaining to the worldwide occurrence of ZIKV-CHIKV coinfection.

Most of the studies that were analysed in this meta-analysis on the coinfection of ZIKV and CHIKV were reported in Brazil. This finding can be attributed to significant occurrences of ZIKV and CHIKV outbreaks that took place in Brazil from 2013 to 2016 [36,61,68,70,95]. The initial documented evidence of ZIKV infection was officially recorded in northeast Brazil in May 2015. However, genomic analyses suggest that the introduction of the virus may have occurred as early as 2013 [96]. On the other hand, the first indigenous occurrences of CHIKV in Brazil were officially verified in Oiapoque, Amapa in September 2014 [97]. Meanwhile, the initial cases of CHIKV infections documented in Rio de Janeiro, Brazil were primarily associated with travel in 2010 [98]. A significant number of outbreaks were then reported between 2014 and 2016 in Brazil. Between the years 2013 and 2015, a total of 223,230 suspected cases of ZIKV and 356,990 possible cases of CHIKV were reported exclusively in Brazil. It is noteworthy that 48% of these cases were subsequently confirmed [95].

Our present analysis revealed a global prevalence of 1.0% [95% CI: 0.7–1.2] ZIKV and CHIKV coinfection. However, the prevalence rates exhibit variation across different countries and regions. The highest prevalence of coinfection was found in Haiti (3.5%), followed by Colombia (2.4%), Mexico (1.8%), and Brazil (1.0%) (Table 2). Episodic occurrence of epidemic/pandemic in these countries over the last decades may contribute to the highest prevalence of ZIKV-CHIKV coinfection. In such areas, the lack of herd and personal immunity would make the population more susceptible to infection, leading to a higher number of cases. It is noteworthy that these countries are located in the tropical and subtropical regions of North and South America and also represent lower- and upper-middle-income economies. Notably, socioeconomic status and climate (tropical and subtropical) are significant contributing factors to the spread and infection of arbovirus including ZIKV and CHIKV [8,9,99]. Even within the same region, different countries might have distinct environmental, societal, and healthcare factors influencing the rate of coinfection between ZIKV and CHIKV. Different strains of zika and chikungunya viruses can coexist, exhibiting different levels of simultaneous circulation and potentials for coinfection. Certain places may possess dominant strains that are less susceptible to the simultaneous infection of persons with both viruses. In addition, countries with poorer healthcare systems or restricted availability of suitable diagnostic tools may underestimate the frequency of coinfection due to misdiagnosis or inadequate reporting. For instance, Mexico and Haiti are both situated on the same continent. However, Mexico possesses a stronger economy and a more developed healthcare system than Haiti. This disparity in resources may contribute to a higher occurrence of ZIKV-CHIKV in Haiti. Overall, climate, the lack of a robust health care system, decreased herd immunity, travel importations, socioeconomic status and frequent occurrence of epidemics or pandemics in these countries can be considered as contributing factors to the variations in prevalence of ZIKV-CHIKV coinfection. Therefore, it is crucial to prioritise vaccination efforts and public health measures in these vulnerable areas to prevent the escalation of outbreaks. Moreover, the process of clinical diagnosis can be challenging, particularly in cases where there is concurrent circulation with other arboviruses, such as ZIKV and CHIKV. Therefore, it is important to establish accurate differential diagnostic methods for the timely identification of these infections. This will help physicians to prescribe the proper medication and reduce disease severity.

After completing a subgroup analysis based on the continents, it was observed that a majority of the reported studies were conducted in South America. This finding indicates that the coinfection of ZIKV and CHIKV is a significant issue in this continent. Although a majority of the studies were reported from South America, the prevalence of coinfection was found to be highest in North America (2.8%). Nevertheless, the prevalence of coinfection we obtained for North America is not representative of the entire region. This is because the combined population of the United States of America (USA) and Canada constitutes around 60% of the total population of North America, and no data were available from these countries to include in our meta-analysis. Additionally, it is possible that the results could be misleading due to the smaller sample size in North America in comparison with South America. While South America offered 36,940 samples, North America only offered 1087 samples. We have also considered age factors in our subgroup analysis. Interestingly, it was found that the prevalence rate of coinfection was three times higher in paediatric patients (2.1%) in comparison with the adult group (0.7%). This finding suggests that children may be more susceptible to coinfections than adults. This is probably because children have an increased tendency to engage in outside activities, hence increasing their susceptibility to encounters with mosquitoes. Additionally, children are less likely to apply insect repellent and wear covered clothing. Furthermore, immature immunity makes them more susceptible to coinfections. There is also a possibility of becoming infected from breastfeeding or via pregnancy. All of these factors may contribute to the higher prevalence of ZIKV-CHIKV coinfection among children. Further research is needed to confirm the underlying causes and develop appropriate preventive measures for paediatric patients.

Egger’s test is frequently employed to evaluate the presence of publication bias in a meta-analysis by examining the asymmetry of the funnel plot and *p*-value. *p*-value of less than 0.05 from the Egger’s test indicates the presence of significant bias. Because the p-value obtained from the Egger’s test is less than 0.001, it is evident that there is a significant bias in the publications included in this meta-analysis. Publication bias can arise from several factors, such as the selective reporting of studies with positive results by the researchers, inclination of academic journals to reject negative findings, inadequate design or implementation of individual studies, and financial support from companies that may be influenced by the outcome of the study [100]. To evaluate the impact of biased studies, a sensitivity analysis was performed. This analysis revealed that excluding small and low-to-moderate quality studies did not significantly alter the overall conclusion about the combined prevalence of ZIKV-CHIKV coinfection. Thus, our estimated pooled prevalence of ZIKV-CHIKV coinfection is robust and reliable.

Considering the significant global impact of ZIKV and CHIKV mono- and dual infections, it is imperative to prioritise collaborative initiatives aimed at tackling this threat. As we do not have specific treatment and preventive vaccines against these arboviruses, it is crucial to implement preventative and control strategies that specifically target the principal vectors (*Aedes albopictus* and *Aedes aegypti*) responsible for their transmission. Several strategies can be implemented to combat the spread of mosquitoes and their associated diseases. Firstly, it is important to eliminate any stagnant water that could potentially serve as breeding grounds for mosquitoes. Secondly, water reservoirs should be covered to prevent mosquitoes from accessing them. Additionally, the use of chemical repellents and insecticides can be employed to deter vectors, as can the implementation of biological control strategies that specifically target mature mosquitoes, larvae, and eggs in order to make them infertile [30]. Moreover, the implementation of advanced monitoring technologies such as drones, satellite images, and artificial intelligence to monitor mosquito populations and forecast epidemics could potentially help in reducing the spread of infection. Novel therapeutic strategies, such as the invention of a multi-viral vaccine, the use of small molecule inhibitors that selectively hinder the replication of ZIKV-CHIKV, and the application of monoclonal antibodies that kill the viruses, could play important roles in the significant reduction of the burden of these coinfections.

## 5. Strengths and Limitations

This study possesses several notable strengths. To the best of our understanding, this study represents the first comprehensive evaluation and synthesis of existing literature pertaining to the worldwide incidence of concurrent zika and chikungunya infections. Moreover, a considerable number of studies were incorporated, resulting in the analysis of data from a substantial number of participants. Moreover, the sensitivity analysis with the exclusion of small and moderate quality studies did not substantially alter the global prevalence derived in this meta-analysis. This observation serves to demonstrate the robustness of findings regarding the global prevalence of ZIKV-CHIKV coinfection. In addition, we were able to consider confounding factors such as age (adult vs. paediatric) and geographical location to conduct the subgroup analysis.

However, it is important to acknowledge that there are certain significant limitations in this meta-analysis. Firstly, this study only included cases of ZIKV-CHIKV coinfection that were confirmed through laboratory testing. Moreover, the reported data were collected from only published research articles. Therefore, the derived pooled estimates may not accurately reflect the actual global prevalence rates, due to issues such as underdiagnosis, misdiagnosis, and underreporting. By examining the relationship between prior exposure, herd immunity, and disease prevalence, we may gain insights into why some regions experience higher rates of infection compared with others. However, we could not include the relationship in our study due to the unavailability of data. Moreover, we could not find data on ZIKV–CHIKV coinfection from the USA and Canada to estimate accurate prevalence in North America. Finally, we were not able to conduct an analysis considering other confounding factors like sex and socioeconomic status, due to the lack of sufficient data.

## 6. Conclusions

This study presents a comprehensive review and meta-analysis that offers statistical evidence for the worldwide prevalence of ZIKV-CHIKV coinfection. The available evidence indicates that the co-occurrence of zika and chikungunya infection is a significant global public health burden with no specific treatment and preventive vaccines. Thus, it is crucial to evaluate both infections during the diagnostic process. Additionally, it is recommended to enhance or implement mosquito vector control measures, including biological and chemical control. In summary, the findings of this study will have practical implications for clinical practice, will influence the development of public health strategies, and will shape the direction of future research endeavours for the generation of effective vaccines and specific antiviral treatments.

## Figures and Tables

**Table 1:** Major characteristics of the included studies.

No.	Study ID (References)	Study Period	Country	Type of Participants	Number of Participants (Female)	Age of the Participant (Mean ± SD, Range) (Years)	Detection Technique	Adult/ Paediatric
1	Souza-Santos 2023 [50]	07/2018 to 10/2018	Brazil	Random selection based on socioeconomic status	2114 (NR)	10–14	Rapid test kit	Paediatric
2	Mac 2023 [32]	12/2020 to 11/2021	Nigeria	All outpatients, pregnant women, and people living with HIV	871 (619)	36.6 (0–80+)	Immunoblot assay	Both adult and paediatric
3	Frota 2023 [51]	02/2018 to 12/2018	Brazil	Women with suspected arbovirus infection	1289 (all female)	15–39	RT-PCR	Both adult and paediatric
4	Khongwichit 2022 [33]	10/2018 to 02/2020	Thailand	Chikungunya suspected patients	1806 (NR)	≤10–>50	RT-PCR	Both adult and paediatric
5	Bailly 2021 [35]	06/2017 to 10/2017	French Guiana	Patients with suspected arbovirus infection	2697 (NR)	34.1 (25–75)	Microsphere immunoassay	Adult
6	Calvo-Anguiano 2021 [52]	04/2015 to 06/2015 and 02/2016 to 03/2016	Mexico	Patients with suspected arbovirus infection	253 (169)	0–>50	RT-qPCR and nested-PCR	Both adult and paediatric
7	Jacques 2021 [31]	10/2018 to 05/2019	Brazil	Pregnant women with obstetric complications	780 (all female)	26.5 ± 3.6	RT-qPCR	Adult
8	Mota 2021 [53]	2016	Brazil	Patients with compatible symptoms of arbovirus infection	182 (131)	40.06 ± 19.86	RT-qPCR	Adult
9	Leonhard 2021 [54]	12/2014 to 02/2017	Brazil	Patients with a suspected preceding arbovirus infection and an acute neurological disease	71 (36)	46 (32–56)	RT-PCR and ELISA	Adult
10	Eligio-Garcia 2020 [55]	02/2019 to 08/2019	Mexico	Asymptomatic pregnant women	136 (all female)	14–43	RT-PCR and ELISA	Adult
11	Ferreira 2020 [56]	12/2014 to 12/2016	Brazil	Suspected arbovirus-associated neurological disease	201 (106)	48 (34–60)	RT-PCR and PRNT	Adult
12	Perisse 2020 [57]	07/2018 to 10/2018	Brazil	Suspected patients with both symptomatic and asymptomatic arboviral infections	2120 (1624)	43.7 ± 21.4	Rapid test kit	Adult
13	Bagno 2019 [58]	NR	Brazil	Pregnant woman and their respective new-borns with symptoms of arboviral infection	193 (NR)	NR	RT-PCR and ELISA	Both adult and paediatric
14	Ball 2019 [59]	05/2014 To 02/2015	Haiti	Acute febrile illness	252 (120)	7.8 ± 4.5	RT-PCR	paediatric
15	de Souza Costa 2019 [60]	2015 to 2016	Brazil	Acute febrile illness	453 (266)	NR	Rapid colorimetric tests and RT-PCR	Both adult and paediatric
16	Silva 2019 [61]	09/2014 to 07/2016	Brazil	Acute febrile illness	948 (NR)	NR	RT-PCR and ELISA	Adult
17	Mercado-Reyes 2019 [62]	10/2015 to 12/2016	Colombia	Patients suspected of arbovirus infection	23,871 (NR)	NR	RT-PCR	Both adult and paediatric
18	Carrillo-Hernandez 2019 [11]	08/2015 to 04/2016	Colombia	Patients with febrile syndrome	157 (103)	26.81 ± 14.54	Conventional PCR and RT-PCR	Both adult and Paediatric
19	de Souza 2018 [63]	2014 to 2015	Brazil	Patients suspected of arbovirus infection	299 (NR)	NR	RT-PCR and ELISA	Both adult and paediatric
20	Leal Azeredo 2018 [64]	02/2016 to 03/2016	Brazil	Patients suspected of arbovirus infection	134 (NR)	NR	RT-PCR and ELISA	Both adult and paediatric
21	Loconsole 2018 [34]	03/2015 to 06/2017	Italy	Vector-borne disease suspected international travellers	156 (77)	33 (median)	ELISA	Adult
22	Mehta 2018 [65]	11/2015 to 06/2016	Brazil	Patients with new neurological conditions associated with suspected ZIKV infection	35 (NR)	NR	RT-PCR	Adult
23	White 2018 [66]	05/2014 to 07/2014	Haiti	Acute febrile illness	100 (NR)	NR	RT-PCR	paediatric
24	Alva-Urcia 2017 [67]	01/2016 to 03/2016	Peru	Acute febrile illness	139 (63)	NR	RT-PCR	Both adult and paediatric
25	Cardoso 2017 [68]	07/2015 to 04/2016	Brazil	Patients suspected of arbovirus infection	58 (NR)	NR	RT-PCR and ELISA	NR
26	Colombo 2017 [69]	01/2016 to 11/2016	Brazil	Patients with suspected zika virus	433 (287)	36.7 ± 16.8	RT-PCR	Both adult and paediatric
27	Cunha 2017 [70]	02/2016	Brazil	Symptoms of arboviral infections	142 (NR)	NR	RT-PCR and ELISA	Both adult and paediatric
28	da Costa 2017 [71]	03/2016 to 05/2016	Brazil	Symptoms compatible with dengue, chikungunya zika virus infection	273 (175)	37 ± NR	Molecular diagnostics and virus discovery methods	Both adult and paediatric
29	Kaur 2017 [72]	08/2016 to 12/2016	India	Suspected chikungunya virus	600 (NR)	35 ± NR	RT-PCR	Both adult and paediatric
30	Magalhaes 2017 [73]	05/2015 to 05/2016	Brazil	Acute febrile patients with arboviral symptoms	263 (NR)	29 (median)	RT-PCR and ELISA	Both adult and paediatric
31	Cabral-Castro 2016 [74]	04/2015 to 01/2016	Brazil	Patients with suspected dengue fever	30 (NR)	NR	RT-PCR	NR
32	Pessoa 2016 [36]	05/2015	Brazil	Suspected dengue patients	77 (52)	NR	RT-PCR and ELISA	Both adult and paediatric
33	Waggoner 2016 [75]	09/2015 to 04/2016	Nicaragua	Suspected arboviral illness	346 (NR)	NR	RT-PCR	NR

RT-PCR: reverse transcription-polymerase chain reaction; ELISA: enzyme-linked immunosorbent assay; NR: not reported; PRNT: plaque reduction neutralisation testing.

**Table 2:** Pooled prevalence of ZIKV-CHIKV coinfection in different subgroups of countries, continents, and age group.

Subgroups	Prevalence of Zika- Chikungunya Coinfection [95% CIs] (%)	Number of Studies Analysed	Total Number of Subjects	Heterogeneity	
				*I* ^2^	*p*-Value
ZIKV-CHIKV coinfection from different countries					
Brazil	1.0 [0.6–1.4]	19	10,003	87%	<0.01
Colombia	2.4 [0.0–7.3]	2	24,024	88%	<0.01
Haiti	3.5 [0.2–6.8]	2	352	50%	0.16
Mexico	1.8 [0.5–3.1]	2	389	0%	0.71
ZIKV-CHIKV coinfection from different regions					
South America	0.6 [0.4–0.9]	24	36,940	85%	<0.01
North America	2.8 [1.5–4.1]	5	1087	43%	0.13
Asia	0.1 [0.0–0.3]	2	2406	0%	0.59
ZIKV-CHIKV coinfection in adult and paediatric					
Adult	0.7 [0.2–1.1]	10	7326	84%	<0.01
Paediatric	2.1 [0.0–4.2]	3	2466	73%	0.002

CIs: Confidence Intervals.

## Data Availability

The data presented in this study are available within the article Supplementary Materials.

## Supplementary Materials

The following supporting information can be downloaded at: https://www.mdpi.com/article/10.3390/diseases12020031/s1, Figure S1: Prevalence of ZIKV-CHIKV coinfection in different countries, continents, and among adult and paediatric patients; Figure S2: Prevalence of ZIKV–CHIKV coinfection excluding small studies and low- and moderate-quality studies; Table S1: Search strategies; Table S2: Quality assessment of the included cross-sectional studies.
