# Supplementary material for: Global Prevalence of Zika and Chikungunya Coinfection: A Systematic Review and Meta-Analysis
Source: Diseases. 2024 Jan 31;12(2):31. doi: 10.3390/diseases12020031 (PMC10888207; doi:10.3390/diseases12020031)
Supplement: Supplementary file 1 [file diseases-12-00031-s001.zip › diseases-2750000-supplementary/Figure S1_Subgroup analyses.pdf]

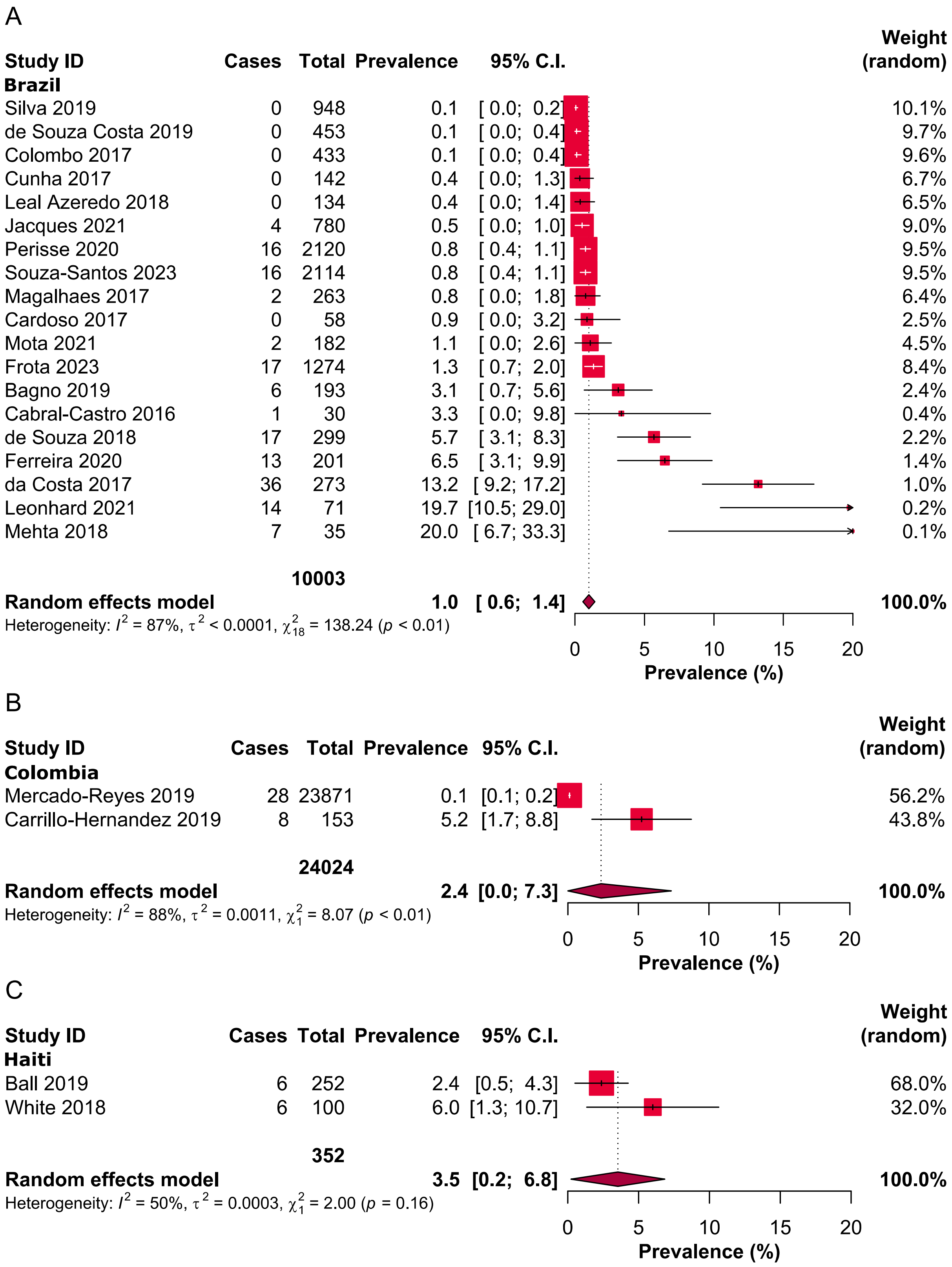
A
Study ID Cases Total Prevalence 95% C.I. Weight (random)
Brazil
Silva 2019 0 948 0.1 [0.0; 0.2] 10.1%
de Souza Costa 2019 0 453 0.1 [0.0; 0.4] 9.7%
Colombo 2017 0 433 0.1 [0.0; 0.4] 9.6%
Cunha 2017 0 142 0.4 [0.0; 1.3] 6.7%
Leal Azeredo 2018 0 134 0.4 [0.0; 1.4] 6.5%
Jacques 2021 4 780 0.5 [0.0; 1.0] 9.0%
Perisse 2020 16 2120 0.8 [0.4; 1.1] 9.5%
Souza-Santos 2023 16 2114 0.8 [0.4; 1.1] 9.5%
Magalhaes 2017 2 263 0.8 [0.0; 1.8] 6.4%
Cardoso 2017 0 58 0.9 [0.0; 3.2] 2.5%
Mota 2021 2 182 1.1 [0.0; 2.6] 4.5%
Frota 2023 17 1274 1.3 [0.7; 2.0] 8.4%
Bagno 2019 6 193 3.1 [0.7; 5.6] 2.4%
Cabral-Castro 2016 1 30 3.3 [0.0; 9.8] 0.4%
de Souza 2018 17 299 5.7 [3.1; 8.3] 2.2%
Ferreira 2020 13 201 6.5 [3.1; 9.9] 1.4%
da Costa 2017 36 273 13.2 [9.2; 17.2] 1.0%
Leonhard 2021 14 71 19.7 [10.5; 29.0] 0.2%
Mehta 2018 7 35 20.0 [6.7; 33.3] 0.1%
10003
Random effects model 1.0 [0.6; 1.4] 100.0%
Heterogeneity: I^2 = 87%, τ^2 < 0.0001, χ^2_{18} = 138.24 (p < 0.01)
0 5 10 15 20
Prevalence (%)
B
Study ID Cases Total Prevalence 95% C.I. Weight (random)
Colombia
Mercado-Reyes 2019 28 23871 0.1 [0.1; 0.2] 56.2%
Carrillo-Hernandez 2019 8 153 5.2 [1.7; 8.8] 43.8%
24024
Random effects model 2.4 [0.0; 7.3] 100.0%
Heterogeneity: I^2 = 88%, τ^2 = 0.0011, χ^2_1 = 8.07 (p < 0.01)
0 5 10 15 20
Prevalence (%)
C
Study ID Cases Total Prevalence 95% C.I. Weight (random)
Haiti
Ball 2019 6 252 2.4 [0.5; 4.3] 68.0%
White 2018 6 100 6.0 [1.3; 10.7] 32.0%
352
Random effects model 3.5 [0.2; 6.8] 100.0%
Heterogeneity: I^2 = 50%, τ^2 = 0.0003, χ^2_1 = 2.00 (p = 0.16)
0 5 10 15 20
Prevalence (%)

D

Study ID	Cases	Total	Prevalence	95% C.I.	Weight (random)
**Mexico**					
Eligio-Garcia 2020	2	136	1.5	[0.0; 3.5]	41.8%
Calvo-Anguiano 2021	5	253	2.0	[0.3; 3.7]	58.2%
		**389**			
**Random effects model**			**1.8**	**[0.5; 3.1]**	**100.0%**

Heterogeneity: $I^2 = 0\%$, $\tau^2 = 0$, $\chi^2_1 = 0.14$ ($p = 0.71$)

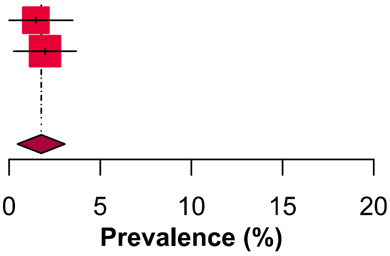

E

Study ID	Cases	Total	Prevalence	95% C.I.	Weight (random)
**South America**					
Silva 2019	0	948	0.1	[0.0; 0.2]	9.7%
de Souza Costa 2019	0	453	0.1	[0.0; 0.4]	8.8%
Colombo 2017	0	433	0.1	[0.0; 0.4]	8.7%
Mercado-Reyes 2019	28	23871	0.1	[0.1; 0.2]	10.0%
Bailly 2021	7	2697	0.3	[0.1; 0.5]	9.5%
Cunha 2017	0	142	0.4	[0.0; 1.3]	4.0%
Alva-Urcia 2017	0	139	0.4	[0.0; 1.4]	3.9%
Leal Azeredo 2018	0	134	0.4	[0.0; 1.4]	3.7%
Jacques 2021	4	780	0.5	[0.0; 1.0]	7.2%
Pessoa 2016	0	77	0.6	[0.0; 2.4]	1.6%
Perisse 2020	16	2120	0.8	[0.4; 1.1]	8.3%
Souza-Santos 2023	16	2114	0.8	[0.4; 1.1]	8.3%
Magalhaes 2017	2	263	0.8	[0.0; 1.8]	3.6%
Cardoso 2017	0	58	0.9	[0.0; 3.2]	1.0%
Mota 2021	2	182	1.1	[0.0; 2.6]	2.2%
Frota 2023	17	1274	1.3	[0.7; 2.0]	6.2%
Bagno 2019	6	193	3.1	[0.7; 5.6]	0.9%
Cabral-Castro 2016	1	30	3.3	[0.0; 9.8]	0.1%
Carrillo-Hernandez 2019	8	153	5.2	[1.7; 8.8]	0.5%
de Souza 2018	17	299	5.7	[3.1; 8.3]	0.8%
Ferreira 2020	13	201	6.5	[3.1; 9.9]	0.5%
da Costa 2017	36	273	13.2	[9.2; 17.2]	0.4%
Leonhard 2021	14	71	19.7	[10.5; 29.0]	0.1%
Mehta 2018	7	35	20.0	[6.7; 33.3]	0.0%
		**36940**			
**Random effects model**			**0.6**	**[0.4; 0.9]**	**100.0%**

Heterogeneity: $I^2 = 85\%$, $\tau^2 < 0.0001$, $\chi^2_{23} = 156.69$ ($p < 0.01$)

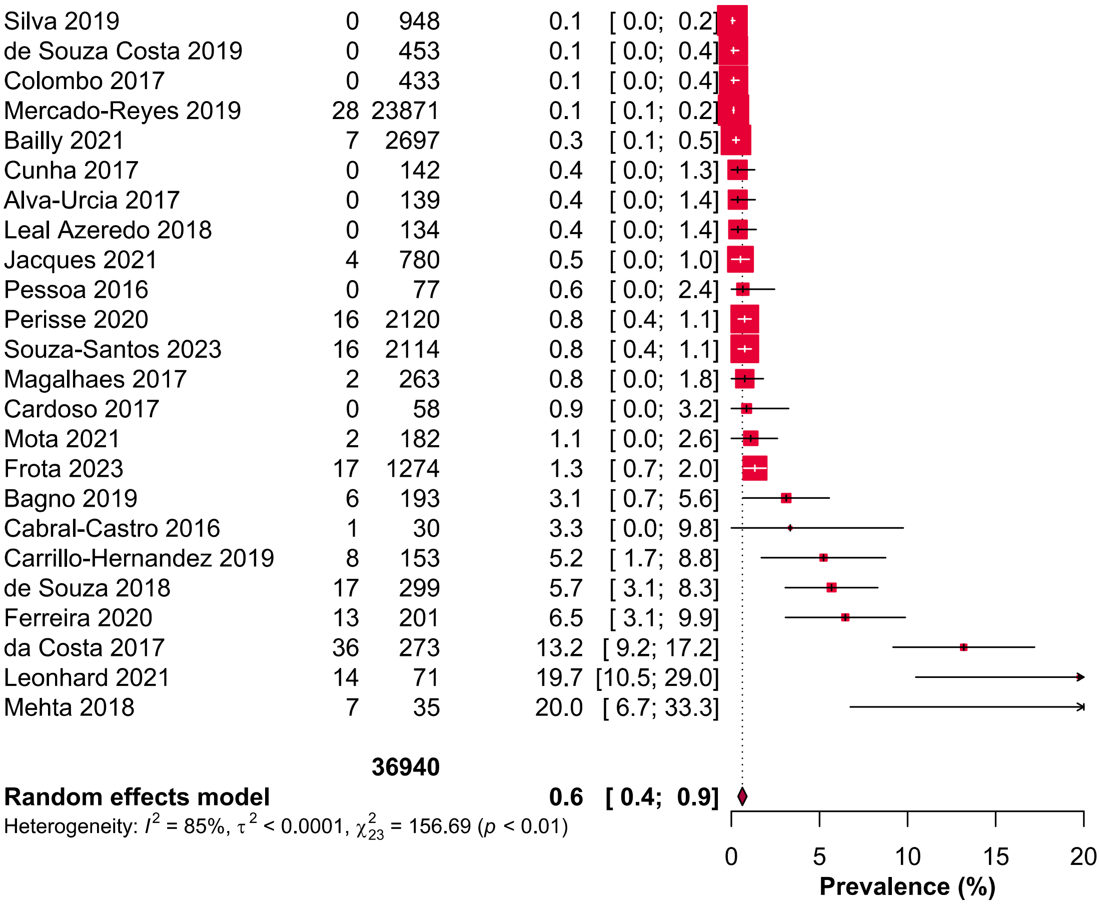

F

Study ID	Cases	Total	Prevalence	95% C.I.	Weight (random)
**North America**					
Eligio-Garcia 2020	2	136	1.5	[0.0; 3.5]	22.4%
Calvo-Anguiano 2021	5	253	2.0	[0.3; 3.7]	26.4%
Ball 2019	6	252	2.4	[0.5; 4.3]	24.1%
Waggoner 2016	16	346	4.6	[2.4; 6.8]	20.3%
White 2018	6	100	6.0	[1.3; 10.7]	6.8%
		**1087**			
**Random effects model**			**2.8**	**[1.5; 4.1]**	**100.0%**

Heterogeneity: $I^2 = 43\%$, $\tau^2 < 0.0001$, $\chi^2_4 = 7.02$ ($p = 0.13$)

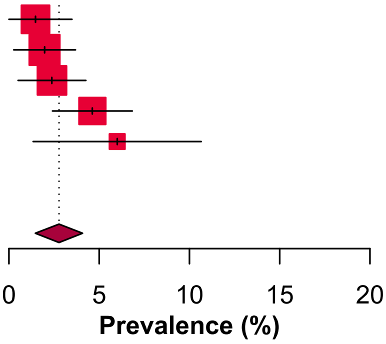

G

Study ID	Cases	Total	Prevalence	95% C.I.	Weight (random)
**Asia**					
Kaur 2017	0	600	0.1	[0.0; 0.3]	39.8%
Khongwichit 2022	3	1806	0.2	[0.0; 0.4]	60.2%
		**2406**			
**Random effects model**			**0.1**	**[0.0; 0.3]**	**100.0%**

Heterogeneity: $I^2 = 0\%$, $\tau^2 = 0$, $\chi^2_1 = 0.30$ ($p = 0.59$)

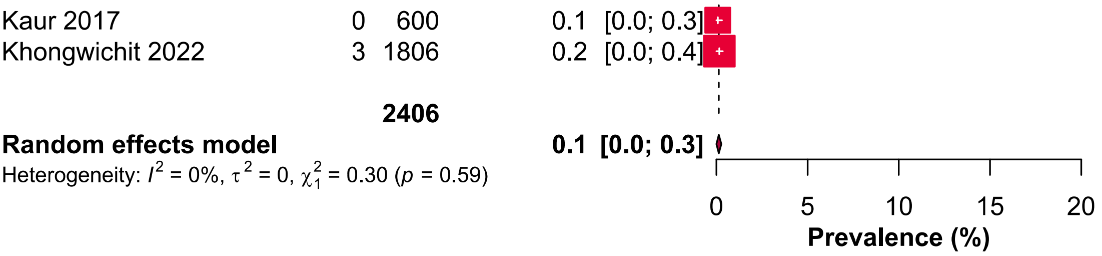

H

Study ID	Cases	Total	Prevalence	95% C.I.	Weight (random)
**Adult**					
Silva 2019	0	948	0.1	[0.0; 0.2]	21.7%
Bailly 2021	7	2697	0.3	[0.1; 0.5]	21.3%
Jacques 2021	4	780	0.5	[0.0; 1.0]	17.4%
Loconsole 2018	1	156	0.6	[0.0; 1.9]	8.1%
Perisse 2020	16	2120	0.8	[0.4; 1.1]	19.3%
Mota 2021	2	182	1.1	[0.0; 2.6]	6.3%
Eligio-Garcia 2020	2	136	1.5	[0.0; 3.5]	4.0%
Ferreira 2020	13	201	6.5	[3.1; 9.9]	1.6%
Leonhard 2021	14	71	19.7	[10.5; 29.0]	0.2%
Mehta 2018	7	35	20.0	[6.7; 33.3]	0.1%
		**7326**			
**Random effects model**			**0.7**	**[0.2; 1.1]**	**100.0%**

Heterogeneity: $I^2 = 84\%$, $\tau^2 < 0.0001$, $\chi^2_9 = 56.37$ ($p < 0.01$)

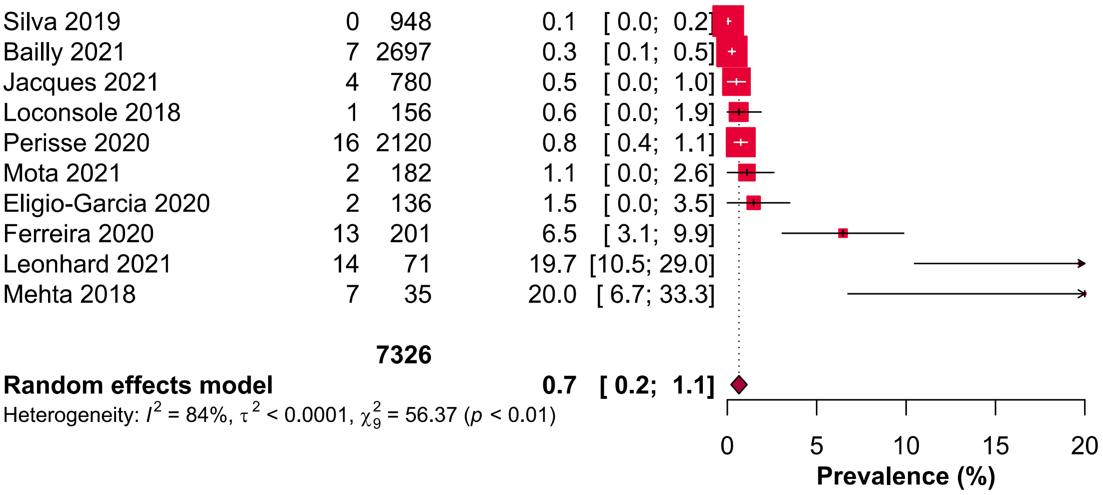

Study ID	Cases	Total	Prevalence	95% C.I.	Weight (random)
**Pediatric**					
Souza-Santos 2023	16	2114	0.8	[0.4; 1.1]	49.7%
Ball 2019	6	252	2.4	[0.5; 4.3]	35.9%
White 2018	6	100	6.0	[1.3; 10.7]	14.4%
		**2466**			
**Random effects model**			**2.1**	**[0.0; 4.2]**	**100.0%**

Heterogeneity: $I^2 = 73\%$, $\tau^2 = 0.0002$, $\chi^2_2 = 7.49$ ($p = 0.02$)

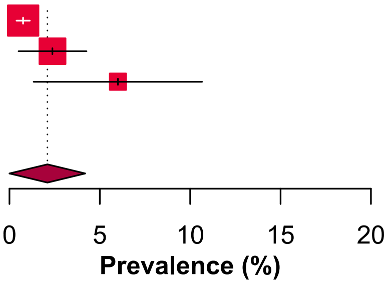

Figure S1. Prevalence of ZIKV-CHIKV coinfection in different countries (A-D), continents (E-G) and among adult and paediatric patients (H-I).
