# Supplementary material for: Global Prevalence of Zika and Chikungunya Coinfection: A Systematic Review and Meta-Analysis
Source: Diseases. 2024 Jan 31;12(2):31. doi: 10.3390/diseases12020031 (PMC10888207; doi:10.3390/diseases12020031)
Supplement: Supplementary file 1 [file diseases-12-00031-s001.zip › diseases-2750000-supplementary/Figure S2_Sensitivity analyses.pdf]

A

Study ID	Cases	Total	Prevalence	95% C.I.	Weight (random)
**Excluding small studies (n<100)**					
Silva 2019	0	948	0.1	[0.0; 0.2]	6.8%
Kaur 2017	0	600	0.1	[0.0; 0.3]	6.6%
de Souza Costa 2019	0	453	0.1	[0.0; 0.4]	6.4%
Colombo 2017	0	433	0.1	[0.0; 0.4]	6.3%
Mercado-Reyes 2019	28	23871	0.1	[0.1; 0.2]	6.9%
Khongwichit 2022	3	1806	0.2	[0.0; 0.4]	6.7%
Bailly 2021	7	2697	0.3	[0.1; 0.5]	6.7%
Cunha 2017	0	142	0.4	[0.0; 1.3]	3.7%
Alva-Urcia 2017	0	139	0.4	[0.0; 1.4]	3.6%
Leal Azeredo 2018	0	134	0.4	[0.0; 1.4]	3.5%
Jacques 2021	4	780	0.5	[0.0; 1.0]	5.6%
Loconsole 2018	1	156	0.6	[0.0; 1.9]	2.8%
Perisse 2020	16	2120	0.8	[0.4; 1.1]	6.2%
Souza-Santos 2023	16	2114	0.8	[0.4; 1.1]	6.2%
Magalhaes 2017	2	263	0.8	[0.0; 1.8]	3.4%
Mota 2021	2	182	1.1	[0.0; 2.6]	2.2%
Frota 2023	17	1274	1.3	[0.7; 2.0]	5.1%
Eligio-Garcia 2020	2	136	1.5	[0.0; 3.5]	1.5%
Calvo-Anguiano 2021	5	253	2.0	[0.3; 3.7]	1.9%
Ball 2019	6	252	2.4	[0.5; 4.3]	1.6%
Bagno 2019	6	193	3.1	[0.7; 5.6]	1.1%
Waggoner 2016	16	346	4.6	[2.4; 6.8]	1.3%
Carrillo-Hernandez 2019	8	153	5.2	[1.7; 8.8]	0.6%
de Souza 2018	17	299	5.7	[3.1; 8.3]	0.9%
White 2018	6	100	6.0	[1.3; 10.7]	0.3%
Ferreira 2020	13	201	6.5	[3.1; 9.9]	0.6%
da Costa 2017	36	273	13.2	[9.2; 17.2]	0.4%
Mac 2023	151	871	17.3	[14.8; 19.9]	1.0%
		**41189**			
**Random effects model**			**0.9**	**[0.6; 1.2]**	**100.0%**

Heterogeneity: $I^2 = 92\%$, $\tau^2 < 0.0001$, $\chi^2_{27} = 342.88$ ($p < 0.01$)

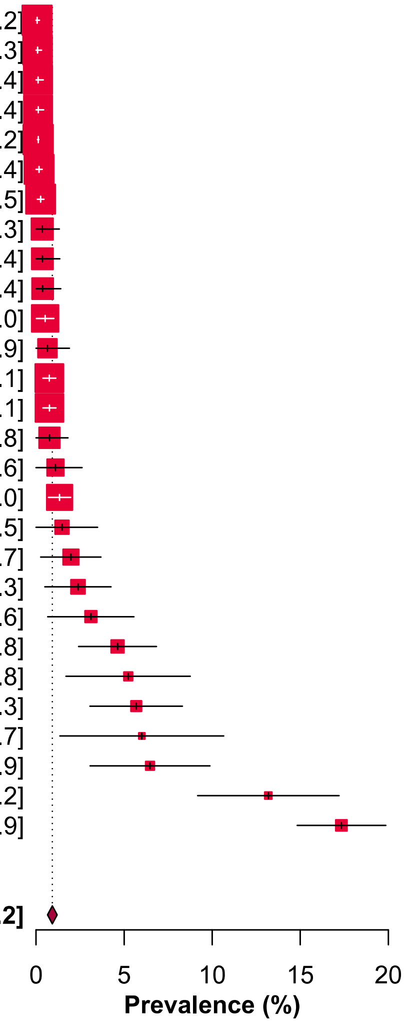

B

Study ID	Cases	Total	Prevalence	95% C.I.	Weight (random)
**Excluding low or moderate quality studies**					
Silva 2019	0	948	0.1	[0.0; 0.2]	12.2%
Kaur 2017	0	600	0.1	[0.0; 0.3]	11.6%
de Souza Costa 2019	0	453	0.1	[0.0; 0.4]	10.9%
Colombo 2017	0	433	0.1	[0.0; 0.4]	10.8%
Mercado-Reyes 2019	28	23871	0.1	[0.1; 0.2]	12.6%
Cunha 2017	0	142	0.4	[0.0; 1.3]	4.8%
Alva-Urcia 2017	0	139	0.4	[0.0; 1.4]	4.7%
Leal Azeredo 2018	0	134	0.4	[0.0; 1.4]	4.5%
Loconsole 2018	1	156	0.6	[0.0; 1.9]	3.4%
Pessoa 2016	0	77	0.6	[0.0; 2.4]	1.9%
Perisse 2020	16	2120	0.8	[0.4; 1.1]	10.3%
Magalhaes 2017	2	263	0.8	[0.0; 1.8]	4.4%
Cardoso 2017	0	58	0.9	[0.0; 3.2]	1.2%
Ball 2019	6	252	2.4	[0.5; 4.3]	1.8%
Bagno 2019	6	193	3.1	[0.7; 5.6]	1.1%
Cabral-Castro 2016	1	30	3.3	[0.0; 9.8]	0.2%
Waggoner 2016	16	346	4.6	[2.4; 6.8]	1.3%
Carrillo-Hernandez 2019	8	153	5.2	[1.7; 8.8]	0.6%
de Souza 2018	17	299	5.7	[3.1; 8.3]	1.0%
White 2018	6	100	6.0	[1.3; 10.7]	0.3%
da Costa 2017	36	273	13.2	[9.2; 17.2]	0.4%
Mehta 2018	7	35	20.0	[6.7; 33.3]	0.0%
		**31075**			
**Random effects model**			**0.6**	**[0.3; 0.8]**	**100.0%**

Heterogeneity: $I^2 = 83\%$, $\tau^2 < 0.0001$, $\chi^2_{21} = 124.57$ ($p < 0.01$)

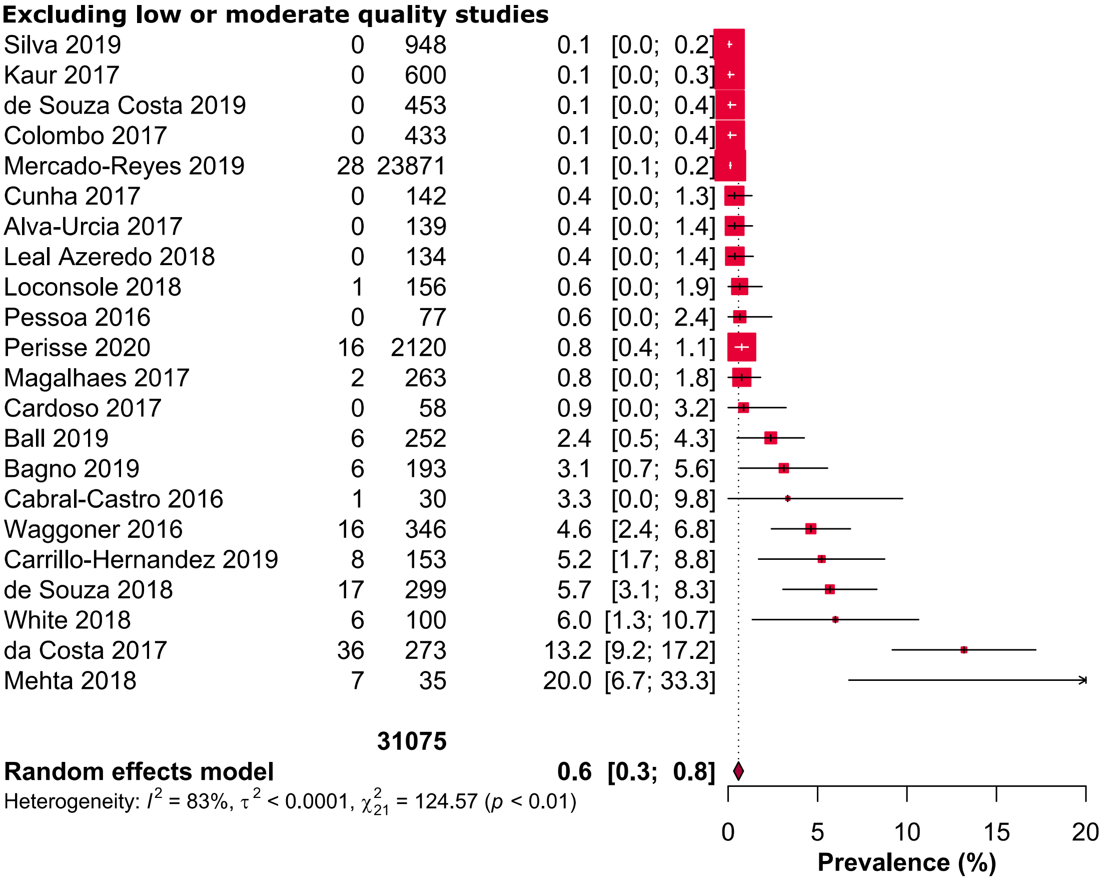

Figure S2. Prevalence of ZIKV-CHIKV coinfection excluding A) small studies and B) low- and moderate-quality studies.
