# Supplementary material for: Global Prevalence of Zika and Chikungunya Coinfection: A Systematic Review and Meta-Analysis
Source: Diseases. 2024 Jan 31;12(2):31. doi: 10.3390/diseases12020031 (PMC10888207; doi:10.3390/diseases12020031)
Supplement: Supplementary file 1 [file diseases-12-00031-s001.zip › diseases-2750000-supplementary/Table S1_Search Strategies.pdf]

Table S1. Search strategies

Databases	Search strategies
**PubMed**	((((Zika[Title/Abstract]) OR ZikV[Title/Abstract]) AND (((Chikungunya[Title/Abstract]) OR CHIK[Title/Abstract]) OR CHIKV[Title/Abstract])) AND (((((((((((((((co-infection[Title/Abstract]) OR co-infections[Title/Abstract]) OR coinfection[Title/Abstract]) OR coinfections[Title/Abstract]) OR co-infected[Title/Abstract]) OR coinfected[Title/Abstract]) OR cocirculation[Title/Abstract]) OR co-circulation[Title/Abstract]) OR concurrent[Title/Abstract]) OR simultaneous[Title/Abstract]) OR simultaneously[Title/Abstract]) OR double-infected[Title/Abstract]) OR dual infection[Title/Abstract]) OR infections[Title/Abstract]) OR arbovirus[Title/Abstract]) OR vector-borne[Title/Abstract])) AND ((((((((prevalence[Title/Abstract]) OR seroprevalence[Title/Abstract]) OR burden[Title/Abstract]) OR epidemiology[Title/Abstract]) OR epidemiological[Title/Abstract]) OR epidemic[Title/Abstract]) OR endemic[Title/Abstract]) OR outbreak[Title/Abstract])
**Scopus**	TITLE-ABS(Zika OR ZikV) AND TITLE-ABS(Chikungunya OR CHIK OR CHIKV) AND TITLE-ABS(co-infection OR co-infections OR coinfection OR coinfections OR co-infected OR coinfected OR cocirculation OR co-circulation OR concurrent OR simultaneous OR simultaneously OR double-infected OR dual OR infection OR infections OR arbovirus OR vector-borne) AND TITLE-ABS(prevalence OR seroprevalence OR burden OR epidemiology OR epidemiological OR epidemic OR endemic OR outbreak)
**Google Scholar**	allintitle:(Zika OR ZikV) (Chikungunya OR CHIK OR CHIKV) (co-infection OR co-infections OR coinfection OR coinfections OR co-infected OR coinfected OR cocirculation OR co-circulation OR concurrent OR simultaneous OR simultaneously OR double-infected OR dual OR infection OR infections OR arbovirus OR vector-borne) (prevalence OR seroprevalence OR burden OR epidemiology OR epidemiological OR epidemic OR endemic OR outbreak)
