# Supplementary material for: Global Prevalence of Zika and Chikungunya Coinfection: A Systematic Review and Meta-Analysis
Source: Diseases. 2024 Jan 31;12(2):31. doi: 10.3390/diseases12020031 (PMC10888207; doi:10.3390/diseases12020031)
Supplement: Supplementary file 1 [file diseases-12-00031-s001.zip › diseases-2750000-supplementary/Table S2_Quality assessment.pdf]

Table S2. Quality assessment of the included cross-sectional studies

Study ID	1	2	3	4	5	6	7	8	Yes (%)
Souza-Santos 2023	U	Y	Y	Y	N	N	Y	Y	62.5%
Mac 2023	Y	Y	Y	Y	Y	N	Y	Y	87.5%
Frota 2023	Y	Y	Y	Y	N	N	Y	Y	75%
Khongwichit 2022	Y	Y	Y	Y	N	N	Y	Y	75%
Bailly 2021	Y	Y	Y	Y	N	N	Y	Y	75%
Calvo-Anguiano 2021	U	Y	Y	Y	N	N	Y	Y	62.5%
Jacques 2021	Y	Y	Y	Y	N	N	Y	Y	75%
Mota 2021	Y	Y	Y	Y	N	N	Y	Y	75%
Leonhard 2021	Y	Y	Y	Y	N	N	Y	Y	75%
Eligio-Garcia 2020	U	Y	Y	Y	N	N	Y	Y	62.5%
Ferreira 2020	Y	Y	Y	Y	N	N	Y	Y	75%
Perisse 2020	Y	Y	Y	Y	Y	Y	Y	Y	100%
Bagno 2019	N	Y	Y	Y	N	N	Y	Y	62.5%
Ball 2019	Y	Y	Y	Y	Y	Y	Y	Y	100%
de Souza Costa 2019	Y	Y	Y	Y	N	N	Y	Y	75%
Silva 2019	U	Y	Y	Y	N	N	Y	Y	62.5%
Mercado-Reyes 2019	Y	Y	Y	Y	N	N	Y	U	62.5%
Carrillo-Hernandez 2019	Y	Y	Y	Y	Y	Y	Y	Y	100%
de Souza 2018	Y	Y	Y	Y	Y	Y	Y	Y	100%
Leal Azeredo 2018	Y	Y	Y	Y	N	N	Y	Y	75%
Loconsole 2018	Y	Y	Y	Y	N	N	Y	Y	75%
Mehta 2018	Y	Y	Y	Y	N	N	Y	Y	75%
White 2018	Y	Y	Y	Y	N	N	Y	Y	75%
Alva-Urcia 2017	Y	Y	Y	Y	N	N	Y	Y	75%
Cardoso 2017	Y	Y	Y	Y	N	N	Y	U	62.5%
Colombo 2017	Y	Y	Y	Y	Y	Y	Y	Y	100%
Cunha 2017	Y	Y	Y	Y	N	N	Y	Y	75%
da Costa 2017	Y	Y	Y	Y	N	N	Y	U	62.5%
Kaur 2017	Y	Y	Y	Y	N	N	Y	U	62.5%
Magalhaes 2017	Y	Y	Y	Y	Y	Y	Y	Y	100%
Cabral-Castro 2016	U	Y	Y	Y	N	N	Y	U	50%
Pessoa 2016	Y	Y	Y	Y	N	N	Y	U	62.5%
Waggoner 2016	Y	Y	Y	Y	N	N	Y	Y	75%

1. Were the criteria for inclusion in the sample clearly defined? 2. Were the study subjects and the setting described in detail? 3. Was the exposure measured in a valid and reliable way? 4. Were objective, standard criteria used for measurement of the condition? 5. Were confounding factors identified? 6. Were strategies to deal with confounding factors stated? 7. Were the outcomes measured in a valid and reliable way? 8. Was appropriate statistical analysis used? Y: Yes, N: No, U: Unclear
